# moco: Fast Motion Correction for Calcium Imaging

**DOI:** 10.3389/fninf.2016.00006

**Published:** 2016-02-16

**Authors:** Alexander Dubbs, James Guevara, Rafael Yuste

**Affiliations:** ^1^Departments of Mathematics and CSE, University of MichiganAnn Arbor, MI, USA; ^2^Neurotechnology Center, Department of Biological Sciences, Columbia UniversityNew York, NY, USA

**Keywords:** Machine Vision Algorithms 150.1135, motion correction, calcium imaging, fourier transform, dynamic programming, mesoscale neuroscience

## Abstract

Motion correction is the first step in a pipeline of algorithms to analyze calcium imaging videos and extract biologically relevant information, for example the network structure of the neurons therein. Fast motion correction is especially critical for closed-loop activity triggered stimulation experiments, where accurate detection and targeting of specific cells in necessary. We introduce a novel motion-correction algorithm which uses a Fourier-transform approach, and a combination of judicious downsampling and the accelerated computation of many *L*_2_ norms using dynamic programming and two-dimensional, fft-accelerated convolutions, to enhance its efficiency. Its accuracy is comparable to that of established community-used algorithms, and it is more stable to large translational motions. It is programmed in Java and is compatible with ImageJ.

## 1. Introduction

Calcium imaging, first used to measure the activity of neurons in the early 1990s (Yuste and Katz, 1991), has been successfully applied throughout the nervous system. It allows us to measure the activity of neurons *in vivo*, using either chemical or genetic calcium indicators, with confocal microscopy, two-photon microscopy, or wearable imaging devices (Grienberge and Konnerth, 2012). As a result, it is an increasingly useful tool for identifying the neural circuits underlying behavior. However, calcium imaging videos have challenging noise properties, including white noise and motion artifacts which must be corrected in a preprocessing step before proper analysis can be undertaken.

Motion correction is the first critical step in the analysis of calcium images. After movies are motion-corrected, ROIs are identified, and time-activity graphs are made from each ROI. If the motion-correction is low-quality, then the time-activity graphs suffer, and the reconstructed networks may have errors. If the motion correction is slow, real time closed loop experiments cannot be done while the mouse is in the microscope.

TurboReg (Thevanaz et al., 1998) is a commonly used algorithm for motion correction. It uses a downsampling strategy, a prerequisite for speed, and a template image, necessary for accuracy. We have independently developed a related method, called *moco* (MOtion COrrector), which adopted both strategies, since correcting one image against the next in the stack results in unacceptable roundoff errors. Other approaches use HMMs (Collman, 2010; Kaifosh et al., 2014) or other techniques (Guizar-Sicairos et al., 2008; Li, 2008; Greenberg et al., 2009; Poole et al., 2015; Ringach, unpublished). Guizar-Sicairos et al. (2008) is the only one similar mathematically to, and may be slightly faster than moco, but it has accuracy problems (see **Figures 2**, **3**).

moco uses downsampling and a template image, and it can be called from ImageJ. However, it is faster than TurboReg (Thevanaz et al., 1998) at translation-based motion correction because it uses dynamic programming and two-dimensional fft-acceleration of two-dimensional convolutions. Guizar-Sicairos et al. (2008) also uses the fft approach with a different objective function that does not require dynamic programming, so our approach is more robust to corrupted data, (see **Figures 2**, **3**). Image Stabilizer is as fast for small images, but is very slow for standard-size images. Running on our own datasets, moco appears faster than all approaches compatible with ImageJ.

moco corrects every image in the video by comparing every possible translation of it with the template image, and chooses the one which minimizes the *L*_2_ norm of the difference between the images in the overlapping region, *D*, divided by the area of *D*. The fact that it is so thorough makes it robust to long translations in the data. More complicated non-translation image warps are usually unnecessary for fixing calcium images, which suffer from spurious translations, which moco corrects, and spurious motion in the Z-direction, something difficult to correct. Our approach also uses cache-aware upsampling: when an image is aligned with the template in the downsampled space, it must be jittered when it is upsampled to see which jitter best aligns with the upsampled template. We do this in such a way that data that is used recently is reused immediately, making the implementation faster. Hence, moco is an efficient motion correction of calcium images, and is likely to become a useful tool for processing calcium imaging movies.

## 2. Mathematical development

Let *a*_*i, j*_, for *i* = 1, …, *m* and *j* = 1, …, *n* be an image in the stack. Let *b*_*i, j*_ be a “template” image against which all other images are aligned, it is typically the first image, a particularly clear image, or the average of the images. We assume *a* is downsampled if it is larger than 256 × 256. We want to pick (*s, t*) such that max(|*s*|, |*t*|) < *w*, where *w* is input by the user, and

$$f_{s,t}=\frac{1}{\text{Area}\left(D_{s,t}\right)}\underset{\left(i,j\right)\in D_{s,t}}{\sum }{\left(a_{i+s,j+t}-b_{i,j}\right)}^{2}$$

is minimal, where *D*_*s, t*_ is the set of ordered pairs of integers (*i*′, *j*′) such that 1 ≤ *i*′ ≤ *m*, 1 ≤ *j*′ ≤ *n*, 1 ≤ *i*′ + *s* ≤ *m*, and 1 ≤ *j*′ + *t* ≤ *n*. If we do this for every image *a* in the stack, we have then motion corrected the video, and we are done, up to a short upsampling step. No ROIs (regions of interest) are used, we use the whole image in every frame in the stack. To upsample, multiply the optimal (*s, t*) by 2 and do a local search to minimize *f*_*s, t*_ on the finer grid. Now,

$$\text{Area}\left(D_{s,t}\right)f_{s,t}=\underset{\left(i,j\right)\in D_{s,t}}{\sum }a_{i+s,j+t}^{2}+\underset{\left(i,j\right)\in D_{s,t}}{\sum }b_{i,j}^{2}$$

$$-2\underset{\left(i,j\right)\in D_{s,t}}{\sum }a_{i+s,j+t}b_{i,j}.$$

The first two sums can be computed via dynamic programming. Let's consider *a* when *s* and *t* are negative. Let

$$g_{s,t}=\underset{\left(i,j\right)\in D_{s,t}}{\sum }a_{i+s,j+t}^{2}.$$

We have that

$$g_{s,t}=g_{s-1,t}+g_{s,t-1}-g_{s-1,t-1}+a_{m+s,n+t}^{2}.$$

Hence, the first two sums can be computed for all (*s, t*) in *O*(*mn*) time, which is unaffected by a constant amount of downsampling. It suffices to compute for all (*s, t*) such that max(|*s*|, |*t*|) < *w*,

$$h_{s,t}=\underset{\left(i,j\right)\in D_{s,t}}{\sum }a_{i+s,j+t}b_{i,j}.$$

Let $\hat{b}$ be *b* rotated 180 degrees. Using MATLAB notation, let

ã=fft2([[a,zeros(m,w)];zeros(w,n+w)]),

$$\tilde{b}=\text{fft}2\left(\left[\left[\hat{b},\text{zeros}\left(\text{m},\text{w}\right)\right];\text{zeros}\left(\text{w},\text{n}+\text{w}\right)\right]\right).$$

Commas denote horizontal concatenation, semicolons denote vertical concatenation, and zeros(*x, y*) is an *x* × *y* matrix of zeros. For equally sized matrices *X*, *Y*, let *Z* = *X* ⊙ *Y* mean *Z*_*i, j*_ = *X*_*i, j*_*Y*_*i, j*_. Then

$$\text{ifft}2\left(ã⊙\tilde{b}\right)$$

is a rearrangement of *h*. Since fft2's are fast, that means *h* can be computed for all (*s, t*) in *O*(*mn*log(*mn*)) time. Hence, after upsampling, the entire video can be aligned in *O*(*mnT*log(*mn*)) time, where *T* is the number of slides in the video. This includes an *O*(*mn*) pixel-by-pixel search for the optimum of *f*_*s, t*_ over all possible (*s, t*) pairs.

After (*s, t*) are chosen to minimize *f*_*s, t*_, they are multiplied by two multiple times to upsample. Every time they are multiplied by 2, *f*_2*s*+*u*, 2*t*+*v*_ are computed for *u, v* ∈{0, −1, 1} to see which *u* and *v* are minimal. These nine evaluations of *f* are done with a cache-aware algorithm for speed. The following flowchart describes the algorithm.


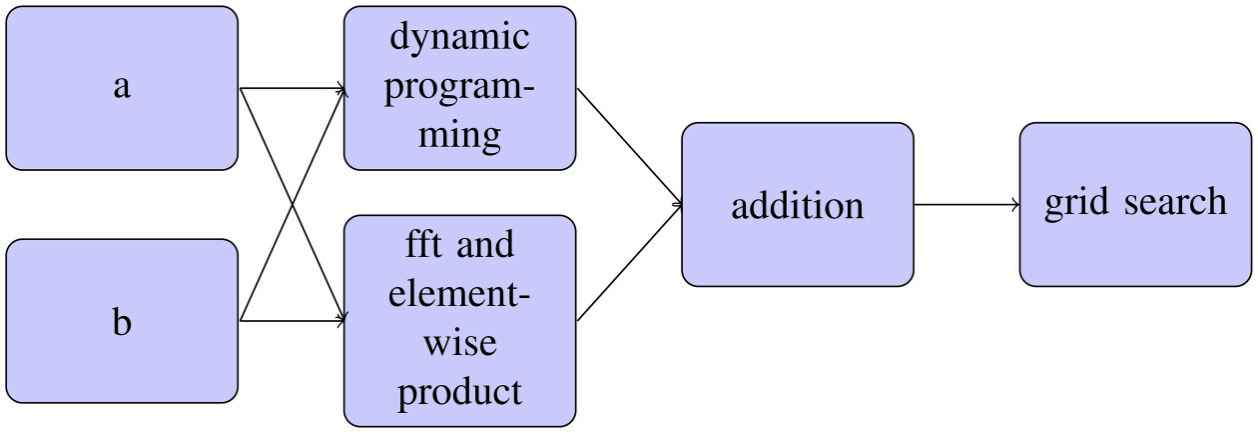


## 3. Results

In Table 1 we compare moco in speed to TurboReg (Thevanaz et al., 1998) on its translation mode, using both the “fast” and “accurate” settings. We also compare it to Image Stabilizer using its default settings (Li, 2008) (it can be made faster by changing the settings but the accuracy is poor). We perform the comparison on several real calcium imaging videos, which we say are *m* × *n* × *T* if they contain *T* slides of size *m*×*n*. If the images are larger than 256 × 256, we downsample once, otherwise, we do not downsample. We have found that dowsampling 3 and 4 times causes severe errors so we avoid those settings. In addition, we have compared moco to TurboReg on synthetic images with severe translational motion artifacts and have found that moco is slightly more accurate. All times are in seconds. The template used for every video is the first image in the video for both moco and TurboReg. moco uses a maximum translation width of min(*m, n*)∕3 in both the *i* and *j* directions.

**Table 1:** **This table compares the speed of moco to competing algorithms**.

**Size**	**moco**	**TurboReg**	**TurboReg**	**Image**
			**(slow)**	**Stabilizer**
512 × 512 × 1500	66 _s_	110 _s_	242 _s_	304 _s_
512 × 512 × 2000	90 _s_	170 _s_	298 _s_	464 _s_
512 × 512 × 6984	288 _s_	632 _s_	1303 _s_	2277 _s_
416 × 460 × 1000	35 _s_	71 _s_	132 _s_	41 _s_
256 × 256 × 2028	84 _s_	121 _s_	154 _s_	34 _s_

**Figure 1:**
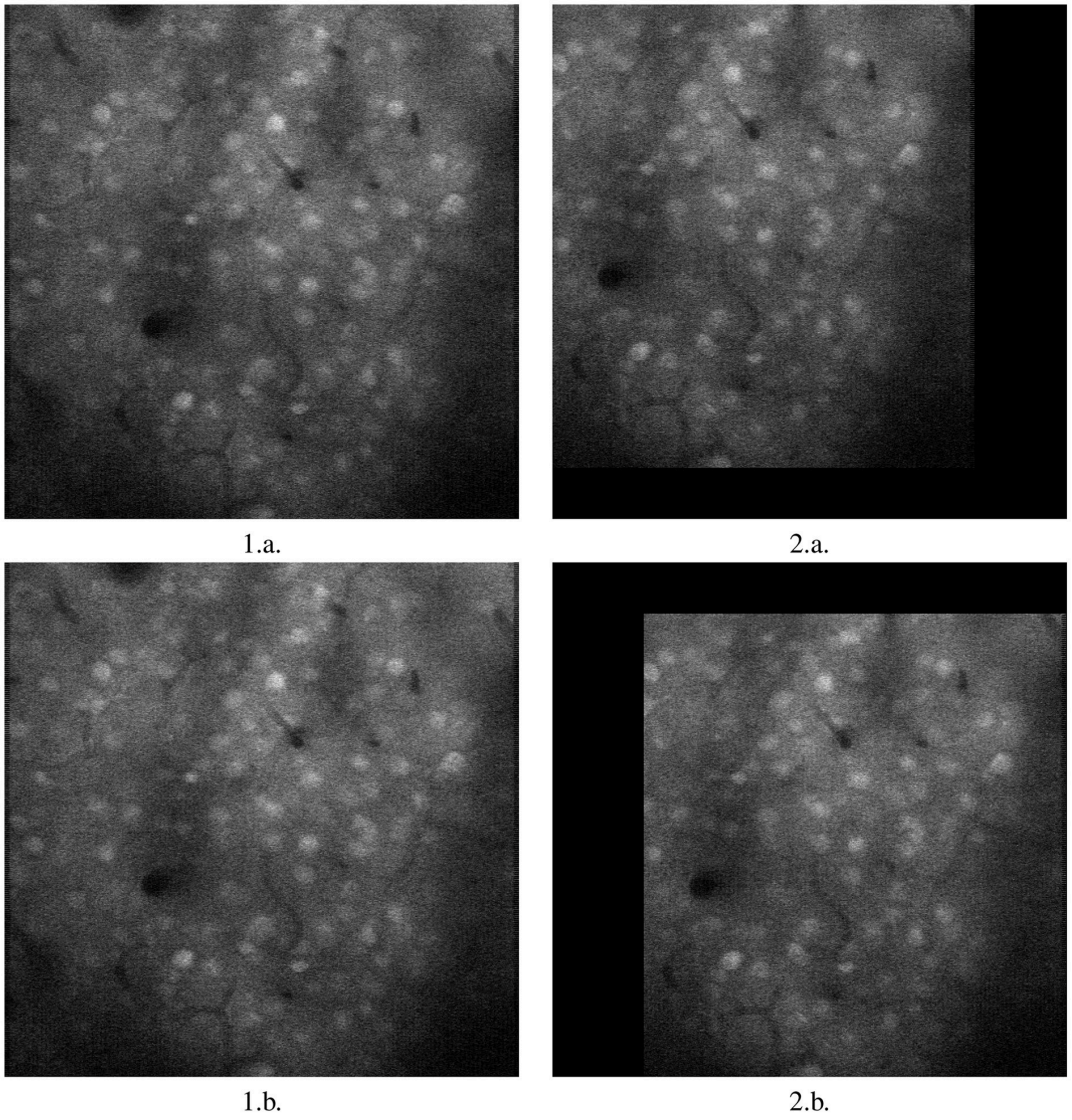
**Image registration with moco**. 1.a. and 2.a. are first two images of a long, badly corrupted video submitted to moco. 1.b. and 2.b. are the two corrected images. Note that 1.a. and 1.b. are identical, since 1.a. is used as the template image. However, 2.b. is registered by moco, moved to the right to overlap 1.b., it matches it almost perfectly except for the non-overlapping black rim. Images are 317.44 × 317.44 mm.

**Figure 2:**
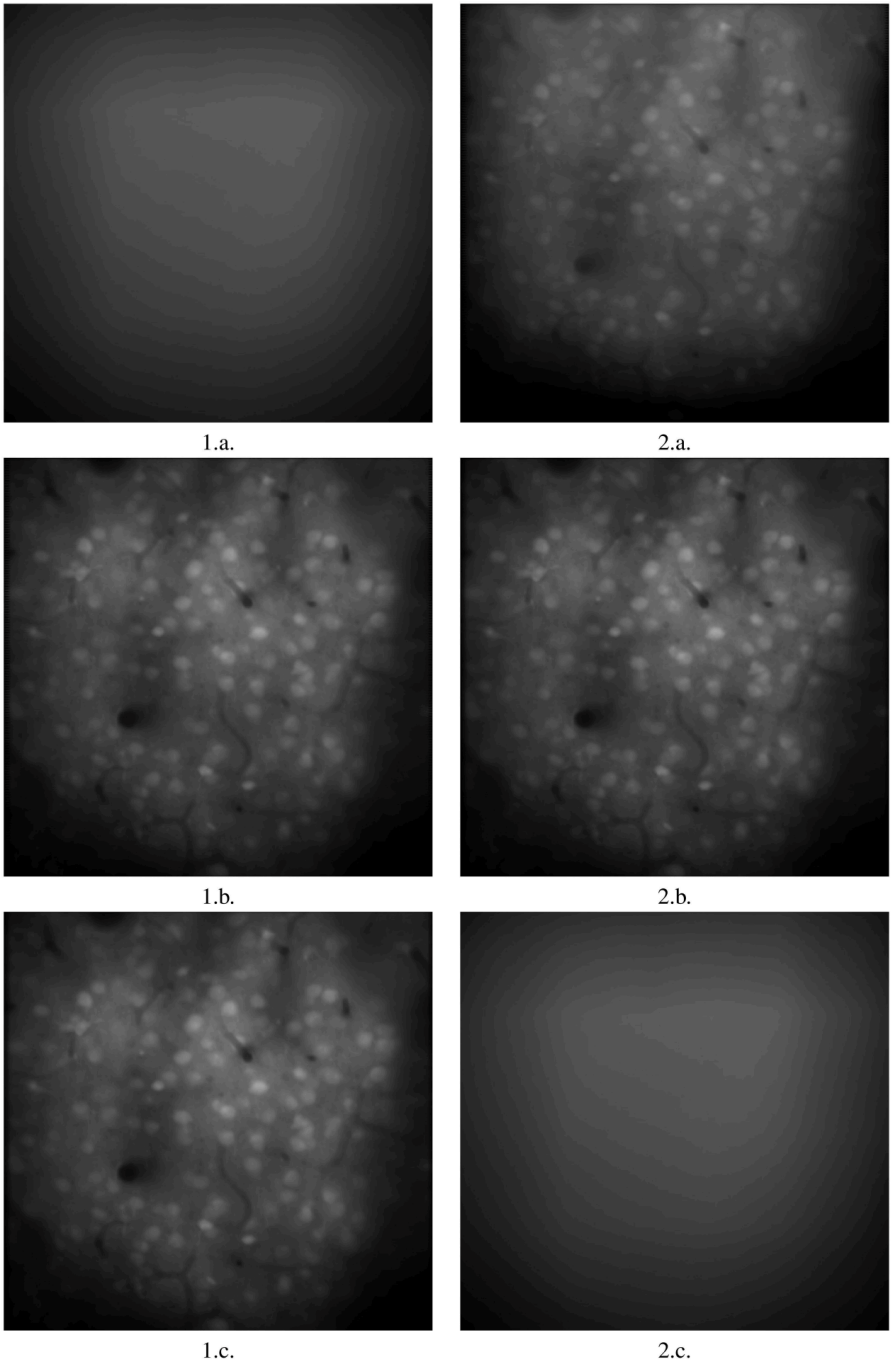
**Image registration with two comparable algorithms**. 1.a. is the mean of all frames in a badly corrupted video. 2.a. is the of corrected video using our implementation of the (Guizar-Sicairos et al., 2008) approach. 1.b. is the mean of the corrected video using moco. 2.b. is the mean of the corrected video using TurboReg (accurate mode), Thevanaz et al. (1998) 1.c. is the mean of the corrected video using TurboReg (accurate mode). 2.c. is the mean of the corrected video using Image Stabilizer. Note that moco and TurboReg have superior performance, as noted by the sharper and brighter appearance of the cell bodies. Images are 317.44 × 317.44 mm.

**Figure 3:**
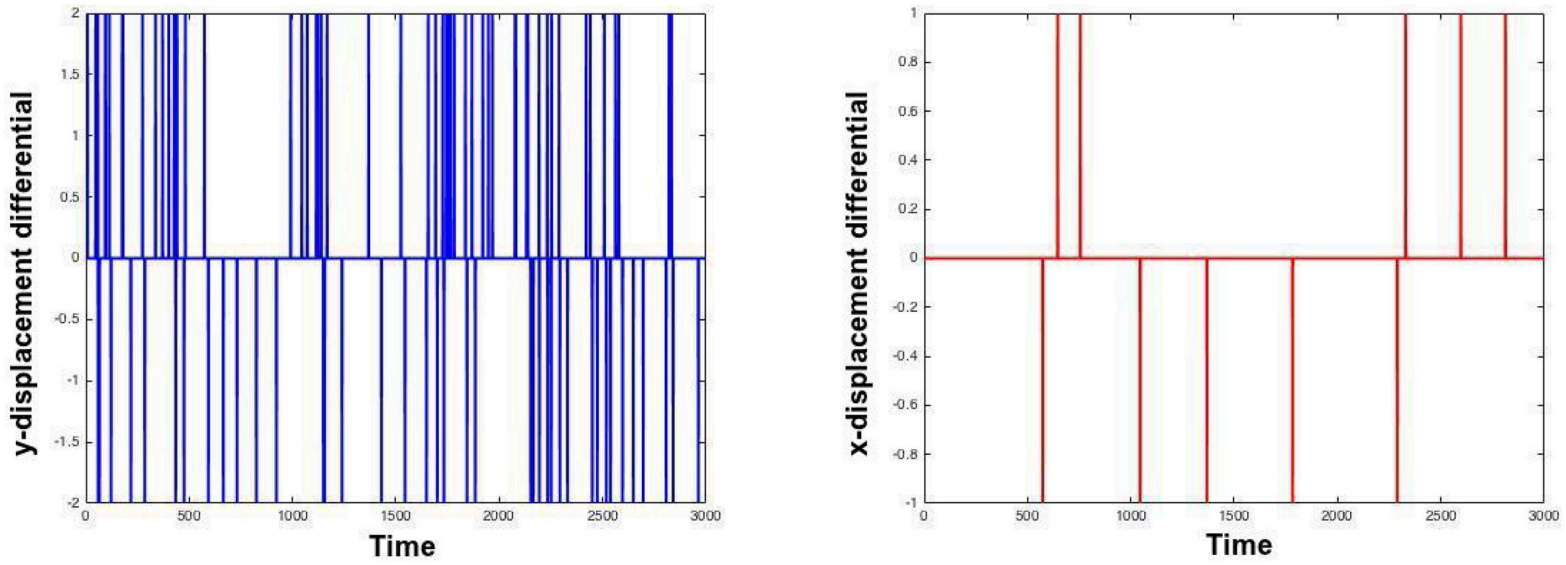
**Analysis of spurious translations by moco and a similar algorithm**. Left and right images *i* right show differences in displacement in the first and second dimensions as a function of time in moco and (Guizar-Sicairos et al., 2008). The video on which they were applied was a real calcium image with added horizontal and vertical spurious translations, to make the task more difficult. moco and (Guizar-Sicairos et al., 2008) generate different translations, and the differences in the translations found are plotted. The left plot shows the y-translations that moco makes minus the y-translations that (Guizar-Sicairos et al., 2008) makes. This difference is typically zero, but there are notable exceptions. The right plot does the same thing for x-translations. Note how moco detects many more translations.

As is clear from the Table 1, moco is faster than its most used current method, TurboReg. It may be marginally slower than (Guizar-Sicairos et al., 2008), but **Figures 2**, **3** prove that a code we have created to have similar results to Guizar-Sicairos et al. (2008) is inaccurate. Figure 1 shows the first two images of a corrupted video on the first row. moco corrections are on the second row. It is clear that moco can fix the image motion, even though the problems with it are severe. Figure 2 shows the mean image from a corrupted video (i.e., add every image in the stack together via matrix addition and then divide the resulting matrix by the number of images in the stack), and the mean image of moco and TurboReg corrections, as well as the correction from our MATLAB version of the (Guizar-Sicairos et al., 2008) algorithm. Note that the (Guizar-Sicairos et al., 2008) algorithm artificially fades the image, indicative of poor alignment, whereas our algorithm and TurboReg are crisp, indicating good alignment. Figure 3 shows the differences in *x* and *y* displacement done by moco and the (Guizar-Sicairos et al., 2008) algorithm in attempt to correct a corrupted calcium imaging video with added spurious translations.

## Author contributions

AD is the first author, RY is the supervisor and corresponding author, JG is a programmer.

### Conflict of interest statement

The authors declare that the research was conducted in the absence of any commercial or financial relationships that could be construed as a potential conflict of interest.
